# Modulating Neurological Complications of Emerging Infectious Diseases: Mechanistic Approaches to Candidate Phytochemicals

**DOI:** 10.3389/fphar.2021.742146

**Published:** 2021-10-26

**Authors:** Sajad Fakhri, Pardis Mohammadi Pour, Sana Piri, Mohammad Hosein Farzaei, Javier Echeverría

**Affiliations:** ^1^ Pharmaceutical Sciences Research Center, Health Institute, Kermanshah University of Medical Sciences, Kermanshah, Iran; ^2^ Department of Pharmacognosy, School of Pharmacy and Pharmaceutical Sciences, Isfahan University of Medical Sciences, Isfahan, Iran; ^3^ Departamento de Ciencias del Ambiente, Facultad de Química y Biología, Universidad de Santiago de Chile, Santiago, Chile

**Keywords:** influenza, dengue, Zika virus, ebola, neurological manifestation, natural products, signaling pathway, pharmacology

## Abstract

Growing studies are revealing the critical manifestations of influenza, dengue virus (DENV) infection, Zika virus (ZIKV) disease, and Ebola virus disease (EVD) as emerging infectious diseases. However, their corresponding mechanisms of major complications headed for neuronal dysfunction are not entirely understood. From the mechanistic point of view, inflammatory/oxidative mediators are activated during emerging infectious diseases towards less cell migration, neurogenesis impairment, and neuronal death. Accordingly, the virus life cycle and associated enzymes, as well as host receptors, cytokine storm, and multiple signaling mediators, are the leading players of emerging infectious diseases. Consequently, chemokines, interleukins, interferons, carbohydrate molecules, toll-like receptors (TLRs), and tyrosine kinases are leading orchestrates of peripheral and central complications which are in near interconnections. Some of the resulting neuronal manifestations have attracted much attention, including inflammatory polyneuropathy, encephalopathy, meningitis, myelitis, stroke, Guillain-Barré syndrome (GBS), radiculomyelitis, meningoencephalitis, memory loss, headaches, cranial nerve abnormalities, tremor, and seizure. The complex pathophysiological mechanism behind the aforementioned complications urges the need for finding multi-target agents with higher efficacy and lower side effects. In recent decades, the natural kingdom has been highlighted as promising neuroprotective natural products in modulating several dysregulated signaling pathways/mediators. The present study provides neuronal manifestations of some emerging infectious diseases and underlying pathophysiological mechanisms. Besides, a mechanistic-based strategy is developed to introduce candidate natural products as promising multi-target agents in combating major dysregulated pathways towards neuroprotection in influenza, DENV infection, ZIKV disease, and EVD.

## Introduction

As emerging infectious diseases, influenza, dengue virus (DENV) infection, Zika virus (ZIKV) disease, and Ebola virus disease (EVD) demonstrate various peripheral and central complications. Studies have shown that neurological manifestations have been a critical part of the aforementioned emerging infections (Billioux et al., 2016), through a direct infection and obliteration of neuronal cells. In this line, glial cells, neurons, and progenitors are major targets of the viruses, leading to less cell migration, neurogenesis impairment, and death (Russo et al., 2017). Among the neuronal complications of emerging infectious diseases, major manifestations are encephalopathy, meningitis, myelitis, stroke, Guillain-Barré syndrome (GBS), radiculomyelitis, meningoencephalitis, memory loss, headaches, cranial nerve abnormalities, tremor, and seizure (Munoz et al., 2018). There are multiple signaling mediators behind the neuronal signs of emerging viral infections, including inflammatory mediators and oxidative pathways. Consequently, interleukins (ILs), interferons (IFNs), toll-like receptor (TLR), nuclear factor-kappa B (NF-κB), mitogen-activated protein kinase (MAPK), inducible nitric oxide synthase (iNOS), Tyro3/Axl/Mer (TAM), and aquaporins (AQP) are pivotal dysregulated factors in the pathogenesis of general complications in emerging infectious diseases towards neuronal manifestations. Additionally, various steps of the virus life cycle are of great importance towards associated neuronal signs (Mohammadi Pour et al., 2019).

Despite advances in revealing the pathophysiology of emerging infectious diseases, underlying neuronal mechanisms require further investigation (Russo et al., 2017). On the other hand, considering multiple dysregulated pathways behind the aforementioned neuronal signs, no specific drug has been found to treat neuronal symptoms of emerging infections. Recent developments in providing novel molecular and cellular mechanisms of virus invasion/replication/proliferation have shown alternative effective and innovative therapeutic strategies (Mohammadi Pour et al., 2019). Phytochemicals are promising multi-target agents with promising antiviral potentials for the treatment of infection. These metabolites are auspicious sources of novel chemical classes of drugs and pharmacological mechanisms with profitable biological activities and health benefits (Naseri et al., 2019). These secondary metabolites have attracted particular attention and have opened a new road in treating neuronal manifestations of infectious diseases by targeting inflammation, oxidative stress, and several other signaling mediators (Fakhri et al., 2020a).

Besides, limited studies reported natural secondary metabolites and candidate phytochemicals as promising agents for the prevention/treatment of emerging infections. This is the first comprehensive review on neurological manifestations of emerging infectious diseases and associated dysregulated pathways as well as the modulatory effects of candidate phytochemicals on the associated signaling pathways.

## Study Design

Scopus, Medline, PubMed, Cochrane, and Web of Science were employed as electronic databases to conduct the comprehensive review. Besides, related articles in other sources were included. Accordingly, keywords (“Influenza” OR “Dengue” OR “Zika” OR “Ebola”) AND (“neurological sign” OR “neurological manifestation” OR “neuron” OR “nerve” OR “CNS” OR “central nervous system” OR “brain” OR “neuropathy” OR “neurology” OR “stroke” OR “multiple sclerosis” OR “encephalitis” OR “encephalopathy” OR “Alzheimer’s disease” OR “Parkinson’s disease” OR “pain” OR “Huntington’s disease” OR “multiple sclerosis” OR “autism” OR “aging” OR “depression”) (title/abstract/keywords) were used. All the phytochemicals possessing both the antiviral and neuroprotective activities within the classes (“Alkaloid” OR “Polyphenol” OR “Flavonoid” OR “Terpenoids”) were also searched in the whole text. Overall, the entire plant-derived secondary metabolites with neuroprotective and antiviral effects, modulating neurological complications of emerging infectious diseases, were included. Data were collected without date and language restrictions until March 2021. The screening procedure of retrieved articles was also performed on the reference citation/lists. Regarding completing the search on electronic databases, hand searching also was provided relying on the authors’ expertise on the neuronal pathophysiological mechanisms of emerging infectious diseases and candidate phytochemicals.

## Neurological Manifestations of Emerging Infectious Diseases

As provided, influenza, DENV infection, ZIKV, and EVD show neurological manifestations passing through multiple signaling pathways.

### Neurological Manifestations of Influenza Virus

Influenza virus, belonging to the RNA viruses in the Orthomyxoviridae family, is categorized into four virus types based on antigenically distinct of A, B, C, and D. However, influenza virus types C and D are not considered as health threats (Francis et al., 2019). In a retrospective study conducted by Takia and coworkers, neurological manifestations of influenza A (H1N1) were reported during the 2019 outbreak (Takia et al., 2020). The neurological manifestations include altered sensorium, cerebrospinal fluid (CSF) pleocytosis, and seizures (Takia et al., 2020). Based on neuroimaging evidence, acute necrotizing encephalopathy, diffuse cerebral edema of childhood, elevation in intracranial pressure (ICP), and acute disseminated encephalomyelitis were also reported as H1N1 neuronal complications (Takia et al., 2020).

Based on the previous reports, all types of influenza, such as seasonal H_1_N_1_ and associated pandemic in 2009, displayed a meaningful impact on both the central nervous system (CNS) and peripheral nervous system (PNS) (Blut, 2009; Paksu et al., 2018). Accordingly, other neurological symptoms in patients with H1N1 are sensory polyneuropathy with flaccid tetraparesis, somnolence, coordination disorder, stupor, confusion, language, and behavior disorders. Besides, disorientation, memory dysfunction, nystagmus, positive Kernig’s sign (Radzišauskienė et al., 2021), febrile convulsion, encephalopathy, acute encephalitis, aseptic meningitis, acute cerebellar ataxia, and myelitis are among major neuronal complications during H1N1. Consequently, GBS, acute mental status change, acute disseminated encephalomyelitis (ADEM), and cerebrovascular illness (e.g., cerebral infarction) are other H1N1-associated neuronal signs (Chen et al., 2020).

In a retrospective study, neurologic complications of the influenza virus were also evaluated. The findings showed that 4% of patients displayed associated neurological manifestations. In this line, the most reiterated complication was influenza-associated encephalitis (IAE) in 65% of patients, and 13% were categorized as having neurological residuals. In addition, 16% showed epileptic seizures, 5% demonstrated acute inflammatory demyelinating polyneuropathy (AIDP), and 14% were classified as having an infection-associated stroke (Mylonaki et al., 2020). One of the most frequent and severe reported neurological manifestations directly related to influenza is IAE, mainly caused by H1N1. However, rare case reports were presented about neurological complications associated with influenza B (Mylonaki et al., 2020). Miller Fischer syndrome, stupor, infection-associated stroke, multiple ischemic strokes at the moment of admittance fever, tetraparesis with right hemispasticity, delirium, and convulsive status epilepticus, consistent with acute hemorrhagic leukoencephalitis, are some other neurologic manifestations of influenza virus (Mylonaki et al., 2020). In one case studied by Mylonaki et al., following developed cerebral edema, secondary cerebral hemorrhage, multiple organ failure (MOF), and cardiopulmonary arrest also occurred (Mylonaki et al., 2020). The neurological complications such as seizures, focal deficit, and altered sensorium are probably associated with febrile seizures, deterioration of preexisting neurological disease secondary to acute illness, sepsis, hypoxia, and MOF. However, a number of patients demonstrate neurological complications in their absence (Jain and Lodha, 2020).

### Neurological Manifestations of DENV

DENV, belonging to the arthropod-borne flavivirus family, is one of the fast-growing tropical infections (Pathak and Mohan, 2019). In Murthy et al*.* study, the neurological involvement of DENV, in the form of atypical cases, was discussed and based on pathogenic mechanisms was categorized into metabolic disturbance (e.g., encephalopathy), viral invasion (e.g., meningitis, encephalitis, myelitis, and myositis), and immune-mediated reactions, inclusive of neuromyelitis optica, myelitis, ADEM, optic neuritis, encephalopathy, neuroophthalmic complications, and GBS (Murthy, 2010). The new classification was categorized into the DENV associated involvement of CNS, PNS, and post-DENV immune-mediated syndromes (Solbrig and Perng, 2015). Panda et al. indicated that DENV encephalopathy, transverse myelitis, and cranial nerve palsies are other neural manifestations of DENV disease. Besides, parkinsonism secondary to DENV infection is of other uncommon neural presentations. In a case study, a 13-year-old premorbidly normal boy was described with bradyphonia, bradykinesia, mask face, and cogwheel rigidity (Panda et al., 2020). In another case report that was conducted by Ho and coworkers, a case of DENV fever with unrelated neuropathies was highlighted. Consequently, mononeuritis multiplex, full resolution of diplopia, and partial resolution of left foot drop were manifested (Ho et al., 2020). In another report, uncommon manifestations of DENV disease were reported by Tun and coworkers, including photophobia, anxiety, irritability, generalized fits, loss of consciousness, confusion, respiratory failure (assisted ventilation), irritability, lethargy, alteration of mental status, and brain stem dysfunction symptoms. Additionally, confusion, neck stiffness, disorientation, hallucinations, transverse myelitis, and numbness in extremities of the upper arm as well as affecting weakness and picking pain were also reported as DENV-neuronal signs (Tun et al., 2020).

### Neurological Manifestations of ZIKV

ZIKV belongs to the RNA virus in the Flaviviridae family, closely related to other flaviviruses, including DENV, West Nile virus, Japanese encephalitis virus, Chikungunya virus, and yellow fever virus (Arora, 2020). Zika infection was closely related to CNS and PNS diseases, in particular stroke or transient ischemic attack, GBS (Ferreira et al., 2020), meningoencephalitis, transverse myelitis (Da Silva et al., 2017), retroorbital pain (Sharma et al., 2020), and myalgia (Bandeira et al., 2020). Evidence has shown a close relationship between Zika infection during pregnancy, congenital abnormalities, and microcephaly (Mlakar et al., 2016). One of the critical impairments of ZIKV in newborns may result in microcephaly and congenital CNS malformation (Pan American Health Organization/World Health Organization (PAHO/WHO), 2016; Kazmi et al., 2020). Of some newborns with congenital ZIKV and normal head circumference, later developed microcephaly is provided (Pereira et al., 2020). A special tropism of ZIKV demonstrates the CNS manifestation such as generating cerebral calcifications, impairing the development of the fetal brain, intrauterine growth restriction, ventriculomegaly, and finally fetal demise in some cases (Ventura and Ventura, 2018; Beckman et al., 2020).

In a report by Pereira et al*.,* some characteristics of congenital Zika syndrome were detected by head computerized tomography (CT) from the junction of white and grey matter (Pereira et al., 2020). The degree of calcifications was variable, from sparse and scant to coalescent and multiple (Pereira et al., 2020). Additionally, cortical dysplasia was identified, in different presentations, from focal and small to diffuse and large lesions in both hemispheres (Pereira et al., 2020). Cortical dysplasia presented as pachygyria or an infinitesimal thin cortex with agyria, closely linked to hydrocephalus or loss of white-matter volume. In a survey by Pereira et al*.*, children demonstrated a reduction in cerebral volume assorting from slight to severe (Pereira et al., 2020). The findings demonstrated cortical dysplasia, subcortical calcifications, and variations in disease involvement of children comprising scant calcification and multiple coalescent foci. Besides, cortical dysplasia was reported to impact both cerebral hemispheres and focal dysplasia in the insular and temporal lobes. Consistently, cortical thickening, diffuse pachygyria, and cerebral parenchymal thinning with agyria, related to obstructive hydrocephalus, are other neuronal complications of ZIKV (Van Den Pol et al., 2017; Pereira et al., 2020).

### Neurological Manifestations of Ebola Virus

The Ebola virus (EBOV), an RNA virus from the Filoviridae family, consists of five species, including *Reston ebolavirus*, *Sudan ebolavirus*, *Taï Forest ebolavirus*, *Bundibugyo ebolavirus,* and *Zaire ebolavirus* (Rojas et al., 2020). Experimental evidence demonstrates two groups of associated neurological manifestations regarding CNS and PNS. Neurological abnormalities referring to EVD (Inan, 2019) associated with the cerebellar pathways, sensory-peripheral nerves, and subcortical structures were observed in most survivors (Bowen et al., 2016).

The CNS neurological manifestations of EVD include seizures, meningoencephalitis (Billioux et al., 2016), encephalopathy (Mobula et al., 2018), respiratory distress (Mobula et al., 2018), hearing loss (Rowe et al., 1999), aural fullness, tinnitus (Mattia et al., 2016), dizziness (Qureshi et al., 2015), depressed mood (Bowen et al., 2016), and coma (Billioux et al., 2016). Similarly, some participants with EVD showed Parkinson’s syndrome with rigidity, shuffling gait, and retropulsion on examination (Bowen et al., 2016). EVD also caused PNS manifestation including malaise, fatigue (West and Von Saint André-Von Arnim, 2014; Fischer et al., 2015), hiccups, headache (Mobula et al., 2018), muscle weakness (Chertow et al., 2016), frontal release signs, myoclonus, asterixis (involuntary movements), hyperreflexia (sustained clonus), myopathy (generalized weakness) (Billioux et al., 2016), retroorbital pain, and arthralgia (Rowe et al., 1999).

In a study by Bowen and coworkers, a certain degree of objective abnormality was observed on neurologic examination of EVD, such as impairments of either saccades or pursuits, tremors, abnormal sensory manifestations or abnormal reflexes, and frontal signs. Consistent focal deficits along with stroke have been also presented in several survivors of EVD, such as those with homonymous hemiparesis, hemianopia, and cranial nerve palsies (Bowen et al., 2016).

Some other neurological complications may appear after Ebola, including memory loss, seizures, cranial nerve abnormalities, headaches, and tremors (Billioux et al., 2016). The other complications of EVD consist of hypomania, decreased short-term memory, mild cerebellar signs, hyperphagia, insomnia, and mild weakness of the lower limbs (Chertow et al., 2016). Multiple magnetic resonance imaging (MRI) of the brain and multiple punctate microvascular lesions were presented in the white matter (Chertow et al., 2016), experimental examination of CSF, and RNA found in patients affected by EVD (Adekanmbi et al., 2021). In a case study analyzed by Chertow et al*.*, 7 months of monitoring the physical and neurological manifestation in EVD showed that individuals presented decreased executive function and chronic fatigue (Chertow et al., 2016). Consequently, altered mental status, ranging from mild confusion to delirium and hallucinations, might also appear but maybe secondary based on variables, including electrolyte abnormalities and shock (West and Von Saint André -Von Arnim, 2014).

## Pathophysiological Mechanisms of Emerging Infectious Diseases

As provided, several dysregulated mechanisms are behind the pathogenesis of emerging infectious diseases.

### Pathophysiological Mechanisms of Influenza Virus

After infection, influenza virus replicates mainly in the epithelium of the respiratory tract. The other cell types, comprising immune cells, can be infected by the virus and involve the production of viral protein. Moreover, the efficiency of viral replication varies between cell types. Among humans, the critical site that the hemagglutinin (HA) molecule is efficiently cleaved and producing the viral particles is the epithelium of the respiratory system. Influenza transmission eventuates from respiratory fomite or aerosols of a susceptible individual that comes into contact with other ones. Investigations in ferrets have shown that the soft palate is the primary source of the influenza virus that could be transmitted between people. Significantly, the soft palate is a rich source of α-2,6-linked sialic acids selected by the HA proteins recently detected in human influenza viruses (Lakdawala et al., 2015). The pathophysiology of influenza virus is caused by lung inflammation and involvement of epithelium of the respiratory system, along with immune responses. The inflammation is able to spread systemically and is being manifested as a MOF (Zangrillo et al., 2013; Kalil and Thomas, 2019).

A case report study observed an increase in the levels of CSF neopterin in viral infection-related acute encephalopathy and encephalitis (Nezu et al., 1999), which is generated by IFN-induced inflammatory stimulation (Fredrikson et al., 1987), as IFNs are generated in the CNS. A significant increase in the levels of tumor necrosis factor-α (TNF-α) in CSF of the children with influenza-related encephalitis and encephalopathy was reported by Togashi (1999). The CNS is an immune-privileged organ, and this is associated with mechanisms in avoiding the related function of the immune system (Fabry et al., 1994).

Reactive astrocytes, microglia, and glial cells have been detected as a pathologic sign of immune-mediated illness of the CNS. CD14 molecules are expressed on the surface of microglia (Becher et al., 1996), could be stimulated by lipopolysaccharide, and produce TNF-α by activated T lymphocytes (Becher et al., 1996). Moreover, the glial cells imitate cytokine signaling of macrophages in the CNS (Kong et al., 1997). When the glial cells become overactivated and the alignment of the cytokine network becomes broken down, the reposition of cytokines is increased in the CNS and leads to cytokine storm in the brain. It is reasonable to hypothesize that the pathophysiology of acute influenza-related encephalitis and encephalopathy is the involvement of the glial cells. It overproduces the inflammatory cytokines, the cytokines are gathered in the brain, and afterward, the brain edema is eventuated; then, the degenerative alterations in the neural cells occur. According to the clinical evidence, influenza virus primarily shows modifications in the mental status and then manifests the rapid systemic alterations described by this hypothesis (Yokota et al., 2000).

From another mechanistic point of view, following the entry of influenza virus, related M2 proton channels are activated by low pH of the associated endosome, thereby acidify the viral interior, and weaken the electrostatic interaction, which allows viral uncoating. In this line, inhibiting the function of M2 ion channel prevents the uncoating of the influenza virus. On the other hand, the HA of the influenza virus binds to receptors with neuraminic acid. The enzymatic activity of neuraminidase (NA) releases viruses by removing neuraminic acids from oligosaccharide chains of receptors. So, NA inhibitors (NAIs) are another class of anti-influenza drugs (Mohammadi Pour et al., 2019).

### Pathophysiological Mechanisms of DENV

Pathogenesis of DENV disease may be directly related to invasion of the CNS, alterations in the metabolism, and autoimmune reaction (Prabhat et al., 2020). Even though the DENV was conventionally deemed to be nonneurotropic, manifestations of viral particles in the cerebrospinal fluid as well as neurological involvement observed with DENV and also damage to the blood-brain barrier (BBB) owing to DENV disease have been contested by these theories (Li et al., 2017). In recent years, several receptors have been identified to be involved in DENV entry, including claudin-1 cell receptors (Che et al., 2013), lectins (Lo et al., 2016), and carbohydrate molecules (Aoki et al., 2006). Among carbohydrate molecules, sulfated polysaccharides, glycosphingolipids (GSL), and glycosaminoglycans (GAGs) are widely expressed coreceptors for DENV entry and efficiency. The highly sulfated GAGs, heparan sulfates (HS), and heparan sulfates proteoglycans (HSPG) are critical for cellular adhesion to extracellular matrix and binding of polypeptide growth factors (Kato et al., 2010) to facilitate binding of the virus to other receptors and then internalization (Laureti et al., 2018). Besides, *in vitro* infection of BV2 microglia cell line with DENV resulted in increased expression of proinflammatory cytokines, including IFN-γ, TNF-α, IL-6, IL-1β, IL-10, and monocyte chemoattractant protein-1 (MCP-1), as well as matrix metalloproteinase- (MMP-) 2 and MMP-9 (Bhatt et al., 2015). Current investigations have also confirmed the role of DENV disease on neuroinflammation. The nonstructural 1 antigen (NS1Ag) is a secreted type of glycoprotein (GP) that initiates the cytokine release and acts as a cofactor for the replication of the RNA virus. The natural killer (NK) cells extremely also have a critical role in the pathogenesis of neurological complications of DENV as demonstrated by NK cell’s early activation and eventually activate T helper cells. These T helper cells are divided and transformed into T helper 17 and T helper 9 cells and elevate subsequent release of proinflammatory cytokines such as IL-4, IL-12, IFN-γ, and transforming growth factor-beta (TGF-β). The proinflammatory cytokines in the next step direct to damage the BBB and afterward promote the entrance of other immune mediators into the brain thereby provoke neuroinflammation (Madi et al., 2014; Prabhat et al., 2020).

### Pathophysiological Mechanisms of ZIKV

ZIKV attacks and remains in some target host cells such as blood, skin, placental cells, neural stem cells, retina, placental progenitor cells, and neural and gonadal tissues (Lee and Shin, 2019; Serman and Gack, 2019). ZIKV demonstrated tropism for neural stem cells and progenitor cells (Tang et al., 2016). As the most critical entry receptors involved in the entrance of flavivirus, some play key roles, including αvβ3 integrins (Fan et al., 2017), C-type lectin receptors (CLR) (Chen et al., 2012b), phosphatidylserine receptors T-cell immunoglobulin, and mucin domain (TIM), as well as TAM (Meertens et al., 2012). Accordingly, ZIKV employs receptor tyrosine kinases to enter the host cells through endocytosis, and facilitating virus replication. Of those receptors, Axl family receptor tyrosine kinases are intensely expressed in numerous cell types of the cerebral cortex (Richard et al., 2017). ZIKV also enters the placenta through Axl-mediated interaction with endothelial cells of the umbilical vein (Poland et al., 2018) and also replicates in other placenta tissues (Quicke et al., 2016). This infection could lead to impairment of the brain/skull development. Besides, neural progenitor cells seem to be direct targets of ZIKV. For instance, neural stem cells and radial glial cells show immunohistochemical evidence of the Axl entry site (Nowakowski et al., 2016; Kakooza-Mwesige et al., 2019). It is able to access host cells *via* Axl receptors existing on the membrane of the host cell.

The Zika life cycle in host cells consists of four stages of viral proteins translation, ZIKV RNA replication, viral particle assembly in the endoplasmic reticulum, and virion release (Kohno et al.). ZIKV encodes three structural proteins, that is, called envelope (E), capsid (C), and precursor membrane (prM), and seven nonstructural proteins, including NS1, NS2A, NS2B, NS3, NS4A, NS4B, and NS5. The structural proteins, E, and prM are used by the virus to attach to the neural cell membrane of the host. The nonstructural proteins and C protein localize to different organelles of the neuron such as Golgi apparatus, nucleus and its nucleoli, and cytoplasm lipid droplet, leading to apoptosis, cell cycle arrest, and cell death (Christian et al., 2019; Lee and Shin, 2019).

ZIKV has been exhibited to bring about DNA damage in host cells by breaking double-strand and keeping the host cell in the S phase, preventing replication. These cellular impacts have been presupposed to direct the death of neural cells of the cortex and cause a deficiently developed brain in fetuses who were infected by ZIKV (Hammack et al., 2019). Moreover, ZIKV is considered to use the cellular defense in humans *via* the nonstructural proteins-mediated interferon antagonism or by reducing the IFN production that is organizing the inhibition of ZIKV replication in human cells (Arora, 2020). Additionally, dysregulation in human embryonic cortical neural progenitor cells (hNPCs) following ZIKV infection revealed a differential expression of genes related to cell cycle dynamics, protein localization, and cell transcription (Tang et al., 2016; Zhang et al., 2016).

### Pathophysiological Mechanisms of EBOV

**FIGURE 1:**
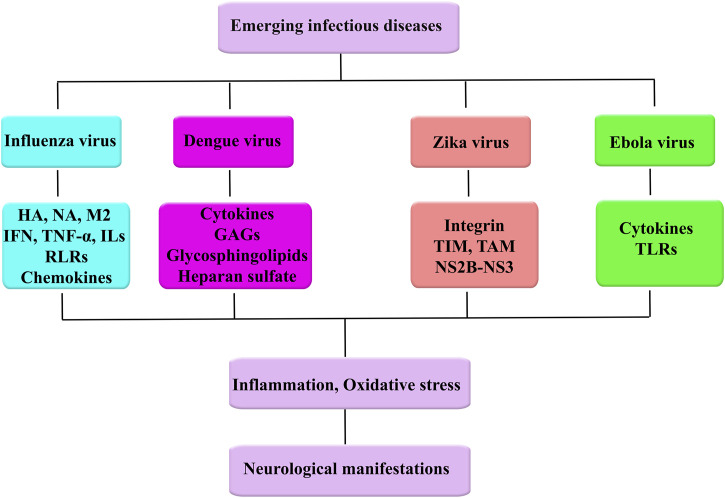
Emerging infectious diseases and associated dysregulated mediators towards neurological manifestations.

EBOV binds to Tim-1 on T lymphocytes and thereby causes robust inflammatory responses referred to as a cytokine storm (Younan et al., 2017). The aforementioned surge in cytokine/chemokine production, as well as dysregulations in autoimmune responses, plays key roles in the pathogenesis of filoviruses complication possessing a near link with acute neurological symptoms (Wong et al., 2014; Bixler and Goff, 2015). Evidence from the West Africa outbreak and experimental studies also indicated that the EBOV might enter the nervous system (De Greslan et al., 2016). However, revealing the exact mechanisms behind the pathogenesis of EVD remained a significant challenge (Kakooza-Mwesige et al., 2019). EVD cell and tissue tropism are principally identified by the EBOV GP_1,2_ attachment factors on the surface of the host cell and GP_1,2_ and intracellular binding to the Niemann–Pick C1 (NPC) intracellular cholesterol transporter 1 receptor (Carette et al., 2011; Côté et al., 2011). Nearly all human cells could get infected, but dendritic cells and mononuclear phagocytes (e.g., microglia, macrophages, and Kupffer cells in the liver) are preliminary EBOV target (Geisbert et al., 1992; Takada et al., 1997; Geisbert et al., 2003a; Ryabchikova and Price, 2004; Bray and Geisbert, 2005; Geisbert et al., 2015). While the preliminary target cells get infected, they likely promote virus dissemination (Schnittler and Feldmann, 1998) and move to the spleen, liver, and regional lymph nodes (Geisbert et al., 2003a). Binding to GP_1,2_ of EBOV causes activation of infected macrophages evaluated by an *in vitro* model (Wahl-Jensen et al., 2005). Moreover, in another *in vitro* model, dendritic cells show a reaction to EVD with partial suppression of histocompatibility complex class II responses, expression of TNF ligand superfamily member 10 (TNFSF10) and tissue factor, reduced secretion of proinflammatory cytokines, and enhanced production of chemokines, for example, IL-8, C-C motif chemokine 2 (CCL2), CCL3, and CCL4 (Hensley et al., 2002; Geisbert et al., 2003b; Bosio et al., 2003; Mahanty et al., 2003; Bosio et al., 2004). Altogether with probable abortive infection (Younan et al., 2019), the TNFSF10 expression and malapropos cytokine responses probably direct to the vast lymphocyte death. This lymphocyte depletion probably attributes to the patients’ susceptibility to EVD towards secondary infections (Geisbert et al., 2000; Geisbert et al., 2003a), hypotension, and ultimately MOF that is general in EVD (Baize et al., 1999; Geisbert et al., 2000; Martines et al., 2015; Jacob et al., 2020). The innate immune systems and systemic adaptive of CNS express pattern recognition receptors (PRR), including TLRs, retinoic acid-inducible gene 1 (RiG-1), and melanoma differentiation-associated protein 5 (MDA-5) that detect viral nucleic acids and promote host antiviral response. Nevertheless, EBOV is recognized and internalized by TLR, MMR, DC-SIGN, CD162, and Scavenger Receptor B as host cell receptors. They also have the potential of escaping immune surveillance by the host systemic and innate immune systems (Denizot et al., 2012). Figure 1 shows emerging infectious diseases and associated dysregulated mediators towards neurological manifestations (Figure 1).

## Conventional Therapeutic Strategies Against Emerging Infectious Diseases: Neuronal Signs and Beyond

To combat the wide complications of emerging viral diseases, conventional strategies are being employed.

### Influenza Virus

Lately, all approved anti-influenza drugs intervene in viral protein function and are categorized to the direct-acting antivirals (DAAs) class. The rapid growth of viral resistance has appeared as the prevailing liability of DAAs, in particular when used against RNA viruses with error-prone polymerases such as the influenza viruses (Hussain et al., 2017; Toots and Plemper, 2020) or the respiratory syncytial virus (RSV) (Devincenzo et al., 2014; Chemaly et al., 2020). For instance, amantadine, adamantanes, and rimantadine were the first approved drugs to treat influenza A virus disease. These inhibitors target the viral M2 ion channel, preventing dissociation of the viral ribonucleoprotein (RNP) genome from the matrix protein by blocking M2-mediated diffusion of protons into virions located in maturing endosomes (Jing et al., 2008; Stouffer et al., 2008). Of some strains of influenza A virus, they are also able to impact virion assembly *via* disturbing M2-mediated pH-equilibration of the organelle Golgi (Jing et al., 2008; Wang et al., 2011).

In spite of the fact that there are no approved biologic drugs for influenza treatment hitherto, neutralizing antibodies (nAbs) have been examined. Therapeutics-based antibodies, in most cases, are well-tolerated and demonstrate desired pharmacokinetic profiles (Ekiert et al., 2012; Tsibane et al., 2012; Laursen and Wilson, 2013). Influenza virus HA was targeted by broadly neutralizing Abs (bnAbs), VIS410, MHAA4548A, and MEDI8852, which are in phase 2 clinical trials. These bnAbs demonstrated some antiviral activities in therapeutic doses and decreased virus replication (Ali et al., 2017; Mcbride et al., 2017; Hershberger et al., 2019). Accordingly, influenza therapeutics currently approved for clinical use are baloxavir, marboxil, oseltamivir, peramivir, and zanamivir (Hayden et al., 1999; Hedrick et al., 2000; Kohno et al., 2010; Heo, 2018).

Immunization based on RNA has appeared as hopeful strategies in comparison with conventional approaches like vaccines (Scorza and Pardi, 2018). Recently influenza vaccines, which have been licensed, exhibited different levels of protection versus seasonal influenza virus strains, though they are insufficient versus pandemic and drifted viruses. Recently, some groups of RNA vaccines showed activity versus influenza virus disease in preclinical models. Moreover, comparative studies displayed the advantages of certain RNA vaccines over the recently utilized inactivated vaccines of influenza virus in animal models. So clinical trials should be initiated and prepare valuable data concerning the translatability of the promising preclinical information to humans (Scorza and Pardi, 2018).

For brain complications of influenza virus, best treatment practice includes initiate antiviral treatment during 48 h of onset (by zanamivir, peramivir, oseltamivir, etc.), administration of a high dose of gamma globulin, and at the same time hormone shock therapy, which decrease the brain metabolism. Plasma exchange therapy is offered when disseminated intravascular coagulation (DIC) and/or MOF exist (Chen et al., 2020). Plasmapheresis and corticosteroids were also administered, but the evidence for their effectiveness is limited (Britton et al., 2017).

### DENV

Currently, a chimeric, attenuated vaccine against DENV (from Sanofi-Pasteur, Dengvaxia) (Hadinegoro et al., 2015) was provided by the vaccine strain of YFV as the backbone with structural proteins of precursor membrane E (prE) and prM of DENV from 1 to 4 serotypes (tetravalent) inserted (Vannice et al., 2018). Whereas this demonstrates another essential milestone in the endeavor to control and prevent flavivirus infections, the differences in immune response based on patient age, viral serotype, and preexisting exposure with DENV are ongoing problems in the usage of this therapeutic agent (Katzelnick et al., 2017; Arredondo-García et al., 2018). This vaccination should not be recommended for individuals who have not been previously infected by DENV (Halstead, 2017; Halstead, 2018). DENV vaccines might have implications for the severity of ZIKV, as it is anticipated that there are conserved antigens and hence cross-reactivity (Bernatchez et al., 2019; Elong Ngono and Shresta, 2019).

### ZIKV

Currently, there is no approved antiviral agent or vaccine against ZIKV disease. The major strategy for controlling ZIKV is suppressing mosquito breeding (World Health Organization, 2016). The common treatment is palliative and consists of fluids intake and rest. Accordingly, paracetamol or acetaminophen is used to reduce fever, myalgia, and headache in patients with ZIKV. The usage of salicylates is not offered in childhood to prevent the probability of Reye’s syndrome [Atif et al., 2016; Centers for Disease Control (CDC), 2016]. Based on Bernatchez and coworkers’ study, the antiviral agents based on their targets are categorized into five groups, including blockers of the entrance stage (e.g., nanchangmycin and ZINC33683341). As second pathway, RNA-dependent RNA polymerase (RdRp) nucleoside analogs like adenosine analog 3 (NITD008) and 7-deaza-2′-C-methyladenosine, sofosbuvir, galidesivir, BCX4430, ribavirin, and emetine are hopeful treatments of ZIKV complications. Protease inhibitors include cn-716 (a boronic acid-containing dipeptide inhibitor), NSC157058, and (5-amino-1-((4-methoxyphenyl) sulfonyl)-1H-pyrazol-3-yl benzoate). Consequently, assembly inhibitors include ST-148 as well as endosomal fusion blockers such as quinacrine, chloroquine, mefloquine, and GSK369796 promise antiviral agents against Zika complications. Finally, nucleoside biosynthesis inhibitors like ribavirin, merimepodib, and methotrexate seem to be promising agents to combat associated manifestations of ZIKV (Bernatchez et al., 2019; Elong Ngono and Shresta, 2019).

## EBOV

There are still no approved vaccines or medications for the EBOV in the world. Prevalent treatment includes monoclonal antibodies, plasma transfusions, small-molecule antiviral compounds, and vaccines (Bishop, 2015). To find effective therapeutics, which specifically block filovirus family members, many investigations have been accomplished to find antiviral agents to intervene with particular stages of the virus entrance (Picazo and Giordanetto, 2015). Conventional associated drugs are predominantly targeting endosomes and interfere with the events like endosomal trafficking, proteolysis of filovirus GP, interactions with NPC1, and finally fusion. Moreover, the number of cathepsin B/L inhibitors (FY-DMK, CA-074, and CID23631927) or nonspecific cysteine protease (leupeptin, E-64) has also been surveyed for their ability to inhibit EVD by *in vitro* models (Chandran et al., 2005; Schornberg et al., 2006; Barrientos and Rollin, 2007; Shah et al., 2010; Gnirß et al., 2012). Currently, another kind of cysteine protease inhibitor, K11777, was detected to inhibit EBOV entry in tissue culture (Zhou et al., 2015). Nevertheless, the impact of plenty of these compounds might not translate to *in vivo* investigations since, as mentioned above, cathepsins B and L are dispensable for *in vivo* EBOV replication. Finally, specific inhibitors for these enzymes might not prove their efficacy versus the filovirus family *in vivo* studies (Marzi et al., 2012). Currently, the antibodies, which bind to GP of the EBOV, have been demonstrated to protect nonhuman primates against lethal EBOV challenge (Qiu et al., 2012; Pettitt et al., 2013; Qiu et al., 2014). KZ52, a neutralized monoclonal antibody, was separated from a human survivor of the EBOV (Maruyama et al., 1999; Lee et al., 2008). KZ52 connects to the base part (hot spot) of the prefusion GP1/GP2 and is caused to neutralize the infection *in vitro* situation (Lee et al., 2008; Dias et al., 2011; Bale et al., 2012). It is worth noting that KZ52 solitary protects guinea pigs and mice against fatal infection but does not protect nonhuman primates (Parren et al., 2002; Rhein and Maury, 2015).

Based on studies, currently, administration of the combination of anti-EBOV GP monoclonal antibodies can protect nonhuman primates against fatal challenges with the EBOV following infection (Qiu et al., 2012; Pettitt et al., 2013; Qiu et al., 2014). One of the effective cocktails is MappBio (MB-003) that consists of the anti-GP antibodies 13F6, 13C6, and 6D8 (Pettitt et al., 2013). The other is Defyrus (ZMAb) that consists of anti-GP antibodies 2G4, 1H3, and 4G7 (Qiu et al., 2012; Audet et al., 2014). Both of these cocktails have been combined to make a drug cocktail of antibodies Z Mapp (2G4, 13C6, and 4G7), which is being accepted as an EBOV therapeutic. The aforementioned antibodies, like 1H3 and 13C6, attach to the glycan cap of GP1, while 2G4 and 4G7 attach to the GP_1,2_ interface in a similar region as 16 F6 and KZ52, and the first monoclonal antibodies in the cocktail efficiently neutralize virus infection *in vitro* model (Audet et al., 2014). According to these investigations, the administration of a combination of antibodies that aims at the GP_1,2_ interface and the glycan cap of GP of EBOV prepares protection *in vivo* situation (Rhein and Maury, 2015).

## Phytochemicals Against Emerging Infectious Diseases: Mechanistic Approaches to Neurological Signs

Emerging infectious diseases are produced by organisms with the capability of transferring from person to person, being a serious threat to human health (Sehgal et al., 2020). Recently scientists investigated the effects of natural products on emerging infectious diseases, including the influenza virus, DENV, Zika, and EBOV (Zheng et al., 2021). Considering the common dysregulated pathophysiological mechanisms of emerging infectious diseases and the capability of some phytochemicals in passing BBB (Fakhri et al., 2020b) urge the need to introduce candidate phytochemicals as potential modulators of peripheral complications, extrapolated to the same dysregulations in CNS.

### Influenza Virus

As previously provided, influenza is accompanied by several neurological manifestations such as encephalitis, encephalopathy (Morishima et al., 2002), fever, cough, sore throat, myalgia, headache, diarrhea, numbness, paresthesia, vertigo, drowsiness, weakness, seizures (Leigh, 1946), AIDP, acute disseminated encephalomyelitis, meningitis, transverse myelitis, and changes of consciousness (Asadi-Pooya et al., 2011).

Evidence has shown that green tea catechins and theanine (as flavonoids) can bind to the HA molecule in the influenza virus, thereby inhibit virus adsorption to the host cell, and lead to the inhibition of influenza infection (Matsumoto et al., 2011). It has been also indicated that green tea extracts increase systemic immunity and can cause inhibition of respiratory tract infection and influenza symptoms in healthy humans; tea catechins include epigallocatechin gallate (EGCG), epigallocatechin, epicatechin gallate, epicatechin, (−)-catechin, and (+)-catechin (Yang et al., 2014). During infection with the influenza virus, EGCG and quercetin increased the level of antioxidant enzymes such as catalase (CAT), glutathione (GSH), and superoxide dismutase (SOD) and thereby suppressed oxidative stress (Kumar et al., 2005; Ling et al., 2012). Consequently, EGCG inhibited the ability of HA protein and viral RNA polymerase as well as NA protein to prevent the cleavage of cell surface sialic acid linked to the virus and inhibited internalization (Kim et al., 2013).

As another phenolic compound, betulinic acid has antibacterial, antimalarial, anti-inflammatory, antihelmintic, antinociceptive, and anticancer activities. It also reduced the levels of inflammatory cytokines, such as IFN-γ, to be a therapeutic agent for the treatment of influenza viral infection through anti-inflammatory properties (Hong et al., 2015). Considering the IFN role in the pathogenesis of the influenza virus, isoquercetin (a flavonol) reduced the levels of IFN-γ and iNOS and scavenged free radicals and interfered with NOS activity towards a decline in neurological manifestations (Kim et al., 2010). Isoquercetin inhibited the replication of influenza A and B at the lowest concentration. Besides, synergistic effects of isoquercetin and amantadine were observed by reducing virus titers, viral replication, pathological changes, and emergence of virus resistance (Kim et al., 2010). Other quercetin derivatives also indicated inhibitory properties in the early stage of influenza infection by inhibiting GP of HA in influenza virus and suppressive effect on virus-induced cellular reactive oxygen species (ROS) generation (Nile et al., 2020) as well as immunomodulation (Wu et al., 2016; Li and Wang, 2019). Several studies suggested that TLR4/NF-κB signaling pathway is an essential inflammatory/oxidative stress response caused by different pathogens (Xu et al., 2017). Accordingly, quercetin declined influenza A virus infection *via* antioxidant potential as well as inhibition of TLR signaling pathway and inhibiting caspase-3 activity (Vaidya et al., 2016). It has been shown that quercetin 3-glucoside is an antiviral agent and indicated more strong anti-influenza A and B activities through blocking the replication and entry of viruses (Nile et al., 2020).

As one class of flavonoids, anthocyanins bind to N1-NA and lead to inhibition of influenza replication (Swaminathan et al., 2014). Baicalin is an anthocyanin with antibacterial, anti-inflammatory, antioxidant, antitumor, antiproliferative, and anticoagulant activity (Xu et al., 2010). It also inhibited the NA activity of influenza A H5N1 and suppressed the level of TLR7, MyD88, NF-κB, and AP-1 (Kannan and Kolandaivel, 2018). Additionally, baicalin suppressed the secretion of TNF-α, IL-1β, IL-6, and IL-8 in H5N1-infected humans and inhibited envelope protein-mediated fusion with chemokine receptors (Hour et al., 2013; Wan et al., 2014). Experimental evidence indicated that baicalin suppressed viral replication in the early phase by triggering macrophage M1 polarization and activating IFN signaling (Geng et al., 2020). Baicalin also inhibited replication of influenza virus A *via* activation of type I IFN signaling by reducing miR-146a (Li and Wang, 2019). Consequently, baicalin downregulated retinoic acid-inducible gene I- (RIG-I-) like receptors (RLRs) signaling pathway (TNF-α, IFN-γ, IL-1β, IL-2, IL-4, IL-5, IL-6, and IL-10) to combat influenza A virus and improved the prognosis (Pang et al., 2018). As an isoflavone, biochanin A affected cellular signaling pathways resulting in reduced virus-induced activation of extracellular signal-regulated kinases 1/2 (ERK1/2), protein kinase B (Akt), and NF-κB. Furthermore, biochanin A inhibited the virus-induced production of interferon gamma-induced protein 10 (IP-10), IL-6, and IL-8, while baicalein inhibited IL-6 and IL-8 production. In their study, baicalein impaired H5N1 virus replication and interfered with the H5N1-induced production of IL-6, IP-10, and TNF-α (Sithisarn et al., 2013). Baicalin inhibits NA (Ding et al., 2014; Jin et al., 2018) and TLR7/MYD88 signaling pathway activation to suppress inflammation in mice infected with influenza A virus (Wan et al., 2014). Evidence suggested that IFN system acts as an important innate antiviral defense mechanism. Recently, it is observed that *Scutellaria baicalensis* Georgi (main constituents including baicalin) has inhibitory activity against various viruses and also can inhibit influenza virus replication affecting inducing IFN-*γ* secretion and also reduced neurological signs (Chu et al., 2015).

Other studies have shown that infection with influenza A virus induces inflammation. Kaempferol has antioxidative and anti-inflammatory effects, thereby declining MAPKs, NF-κB, TNF-α, IL-1β, and blocked ROS generation (Dong et al., 2014; Zhang et al., 2017a). Kaempferol and some other flavonoids have also shown interruption in the influenza life cycle. *In vitro* posttreatment with kaempferol, quercetin, and catechin hydrate noticeably suppressed viral levels of M2 mRNA/protein. *In silico* analysis also found that the aforementioned phytochemicals inhibited influenza A virus-induced replication and autophagy (Choi et al., 2019). Additionally, kaempferol has shown inhibitory activity against influenza and other viruses through reducing EV71 activity and thereby suppressed viral protein translation (Tsai et al., 2011).

Experimental evidence indicated that apigenin (a flavone) suppresses the expression of RIG-1 and also leads to the reduction of IFNs and cytokines in influenza virus infection/replication and associated apoptosis (Xu et al., 2020). Resveratrol as another polyphenol inhibited influenza A virus replication and thereby remarkably improved survival and declined damage (Palamara et al., 2005). Resveratrol can cause inhibition of cellular protein kinase (PKC)/MAPK signaling pathway and reduce virus replication, to be a useful candidate for the treatment of neurological signs (Palamara et al., 2005; Kim et al., 2010). Resveratrol showed a direct inhibition of influenza replication and *via* TLR-9-induced IFN-β production (Lin et al., 2015; Xiao et al., 2015). Based on molecular docking reports, resveratrol derivatives are potential antiviral compounds for developing influenza treatment *via* NA inhibition. Accordingly, resveratrol and catechin 3-*O*-gallate showed an inhibitory effect against NAs activity, with IC_50_ values of 129.8 and 21.3 µM, respectively (Chen et al., 2012a). Considering the results of a docking study, resveratrol and its derivatives (as natural polyphenol) can inhibit the replication of influenza A virus, as well as inhibited intracellular pathways c-Jun N-terminal kinase (JNK) and p38MAPK in the regulation of viral ribonucleoprotein complex (Fioravanti et al., 2012; Li et al., 2015). Evidence has shown that resveratrol and other derivatives have antiviral and antioxidant activities with different mechanisms, such as the inhibition of viral protein synthesis or transcription and modulating viral-related gene expressions or signaling pathways in host cells as it could be useful for the treatment of DENV (Han et al., 2017).

As a triterpene saponin, glycyrrhizin has shown anti-inflammatory and antiviral activities (Finney and Somers, 1958). It declined the level of p38, JNK, and NF-κB (Michaelis et al., 2011), increased NK cell activity, and induced IFN production by T cells (Itoh, 1983). Experimental results indicated that mice receiving lethal doses of the virus survived when treated with glycyrrhizin (Utsunomiya et al., 1997).

As developed, after influenza virus infection, the levels of cytokines were increased, of which geniposide (an iridoid glycoside) significantly inhibited the level of TNF-α, IFN-γ, and IL-6 (Zhang et al., 2017b). Consistently, berberine as a natural alkaloid compound has shown that antioxidant, anti-inflammatory, and anti-influenza effects also inhibited cytopathogenesis and NA activity as well as p38, caspase-3, and NF-κB (Enkhtaivan et al., 2017). It also significantly inhibited the expression of TNF-α and prostaglandin E2 (PGE2) (Cecil et al., 2011). Liu et al*.* observed that berberine inhibited NLR family pyrin domain containing 3 (NLRP3) inflammasome activation in the influenza virus as well as a decline in ROS generation (Liu et al., 2020). In this line, alkaloids also inhibit the function of HA, NA, and M2 in the structure of the influenza virus (He et al., 2013).

Li et al. indicated that aloe emodin (an anthraquinone) binds to virus envelope and leads to the inhibition of influenza A replication and regulated the level of IFN-γ, IFN-β, and double-stranded-RNA-activated protein kinase in influenza A virus (Li et al., 2014). Dai et al. observed that emodin remarkably inhibits the influenza virus through suppressing the expressions of p38/JNK MAPKs, NF-κB, TLR2, TLR3, TLR4, TLR7, MyD88, and TRAF6, as well as increasing nuclear factor erythroid 2-related factor 2 (Nrf2), heme oxygenase-1 (HO-1), SOD, CAT, and glutathione peroxidase (GPx) (Dai et al., 2017).

As another phytochemical, carvacrol (a phenol) has shown anti-inflammatory, antiviral, and antioxidant effects, with inhibitory effects on influenza virus infection. Carvacrol acts through the regulation of the IFN-γ pathway, modulation of IL-6, IL-17, TGF-β, IL-4, and IL-10, and reduction of TLR7, myeloid differentiation primary response 88 (MyD88), interleukin-1 receptor-associated kinase 4 (IRAK4), TNF receptor-associated factor 6 (TRAF6), and NF-κB (Li et al., 2018; Mahmoodi et al., 2019). In this line, cinnamaldehyde has different biological activities, for instance, antibacterial activity, induction of apoptosis through ROS, and inhibition of NOS (Hayashi et al., 2007).

It has also been shown that quercetin, diosmetin, eriodictyol, kaempferol, and isorhamnetin (Dayem et al., 2015) inhibited influenza infection early phase *via* interacting with the HA type 2 subunit of the influenza HA protein. In this line, the inhibitory effect of quercetin against both H1N1 and H3N2 virus leads to the inhibition of virus replication towards the reduction of neurological manifestations (Mehrbod et al., 2021). In another study, isorhamnetin 3-glucoside and quercetin 3-rutinoside revealed higher NAI activity in a dose-dependent manner. A molecular docking study showed that flavonol glycosides have higher binding actions towards influenza polymerase membrane GP towards anti-influenza activity (Kim et al., 2019).

The flavonoids extracted from *Mosla chinensis* Maxim., common name *Moslae Herba* (MHF), have anti-inflammatory, antioxidant, and antiviral effects of inhibiting TLR7, RIG-1, and AQP5 in the alveolar epithelial cells. It also inhibited the influenza virus and neurological symptoms (Yu et al., 2020). Plants rich in caffeic acids, chlorogenic acids, and related derivatives have shown antiviral effects against NA of influenza virus. However, to escape from gut microbiota metabolization, novel delivery systems are recommended (Karar et al., 2016). The flavonoids quercetin, naringenin, catechin, hispidulin, luteolin, chrysin, vitexin, and kaempferol have the potential for developing novel drugs for controlling influenza, which may help to overcome the clinical challenge of the H1N1 strain (Sadati et al., 2019). Kaempferol derivatives, luteolin, quercetin 3-sophoroside, and chelianthifoline show *in vitro* antiviral activity with IC_50_ values ranging from 10.7 to 33.4 µM in comparison to zanamivir 58.3 µM (Lee et al., 2016). These compounds could directly affect the virus itself and inhibit H5N1 viral replication by maintaining cellular redox equilibrium in host cells and blocking the nuclear-cytoplasmic translocation of the viral ribonucleoproteins. They also reduced the expression of late viral proteins related to the inhibition of PKC activity and its dependent pathways. The aforementioned secondary metabolites also showed the potential of downregulating proinflammatory cytokines and protecting organs from the virus- and cytokine-induced oxidative stress by supplying and maintaining sufficient levels of exogenous and endogenous antioxidants (Friel and Lederman, 2006).

Overall, phytochemicals have shown the potential of being used against neurological pathophysiological mechanisms of the influenza virus by targeting the components of the viral life cycle and signaling mediators (Table 1).

**TABLE 1:** Preclinical evidence on the use of candidate phytochemicals against influenza virus and related neuropharmacological effects.

Compounds	Classification	Type of study	Mechanisms	References
Apigenin	Flavone	*In vivo*	↓RIG-I expression	Xu et al. (2020)
			↓IFN and cytokines	
			↓influenza-induced apoptosis	
			↓virus replication	
Aloe emodin	Anthraquinone	*In vivo*	↓replication; regulating the level of IFN-γ, IFN-β and PKR ↓p38/JNK MAPKs, NF-κB, TLR2, TLR3, TLR4, TLR7, MyD88, and TRAF6	Li et al. (2014)
			↑Nrf-2, HO-1, SOD, CAT, and GPx	
Anthocyanins	Anthocyanins	*In silico*	Binds to N1 NA and leads to inhibition of influenza replication	Swaminathan et al. (2014)
Betulinic acid	Triterpenoid	*In vivo*	↓inflammatory cytokines	Hong et al. (2015)
Baicalin	Flavone	*In vivo*	↓NA activity of influenza A H5N1 and Env protein-mediated fusion with chemokine receptors	Xu et al. (2010)
			↓TLR7, MyD88, NF-κB, AP-1, TNF-α, IL-1β, IL-6, and IL-8	Pang et al. (2018)
			↓virus replication *via* activation of type I IFN signaling	Li and Wang, (2019)
			↓miR-146a	
			↓RIG-I-like receptors (RLRs)	
			↓IFN-γ, TNF-α, IL-1β, IL-2, IL-4, IL-5, IL-6, and IL-10	
Biochanin A	Isoflavone	*In vivo*	↓virus-induced activation of Akt, ERK 1/2, and NF-κB	Sithisarn et al. (2013)
			↓inhibited the virus-induced production of IL-6, IL-8, and IP-10; interfered with the H5N1-induced production of IL-6, IP-10, and TNF-α	
Berberine	Alkaloid	*In vivo*	↓cytopathogenic effects and NA activity	Enkhtaivan et al. (2017)
			↓p38, caspase-3, and NF-κB	
			↓TNF-α and PGE2	
			↓NLRP3 inflammasome activation	
			↓ROS generation	
Carvacrol	Phenol	*In vivo*	Modulation of the level of IL-6, IL-17, TGF-β, IL-4, and IL-10	Li et al. (2018)
			↓TLR7, MyD88, IRAK4, TRAF6, and NF-κB	Mahmoodi et al. (2019)
Catechins	Flavan-3-ol	*In vivo/in silico*	↓virus adsorption	Matsumoto et al. (2011)
			↓M2 viral mRNA synthesis and M2 protein expression	Choi et al. (2019)
			↓H1N1 NA	Chen et al. (2012a)
Chelianthifoline	Alkaloid	*In vivo*	↓virally induced cytopathic effect	Lee et al. (2016)
EGCG	Catechins	*In vivo*	↑CAT, GSH, GLU, and SOD	Kumar et al. (2005)
			↓HA protein and viral RNA polymerase NA protein	Ling et al. (2012)
Emodin	Anthraquinone	*In vivo*	↓virus replication directly and *via* TLR-9-induced IFN-β production	Lin et al. (2015)
Glycyrrhizin	Glycosylated saponin	*In vivo*	↓p38, JNK, and NF-κB	Michaelis et al. (2011)
			↑NK cell activity and IFN production by T cells	
Geniposide	Iridoid glycoside	*In silico*	↓TNF-α, IFN-γ, and IL-6	Zhang Y. et al. (2017)
Isorhamnetin 3-glucoside	Flavonoid	*In silico*	Anti-influenza activity and cyclic peptides with anticancer activities	Kim et al. (2019)
Isoquercetin	Flavonoid	*In vivo*	↓replication virus	Kim et al. (2010)
			↓virus titers	
Kaempferol	Flavonol	*In silico*	↓MAPKs, NF-κB, TNF-α, and IL-1β	Dong et al. (2014)
			↓ROS generation	Zhang R. et al. (2017)
			↓M2 viral mRNA synthesis and M2 protein expression	Choi et al. (2019)
Kaempferol 3-sophoroside, kaempferol 3-neohesperidoside, kaempferol 3-sambubioside, and kaempferol 3-glucoside	Flavonol	*In vivo*	↓severity of the virally induced cytopathic effect	Lee et al. (2016)
Luteolin	Flavonol	*In vivo*	↓severity of the virally induced cytopathic effect	Lee et al. (2016)
Quercetin 3-sophoroside	Flavonol	*In vivo*	↓severity of virally induced cytopathic effect	Lee et al. (2016)
Quercetin	Flavonol	*In vivo/in silico*	↓TLR signaling pathway	Vaidya et al. (2016)
			↓caspase-3	Choi et al. (2019)
			↓M2 viral mRNA synthesis and M2 protein expression	Nile et al. (2020)
			↓GP and HA	Friel and Lederman, (2006)
			↓H5N1 viral replication	
			↓nuclear-cytoplasmic translocation of the viral ribonucleoproteins	
			↓proinflammatory cytokines	
			↓oxidative stress by supplying and maintaining sufficient levels of exogenous and endogenous antioxidants	
Quercetin 3-rutinoside	Flavonol	*In silico*	↓NA activity and have higher binding activities towards influenza polymerase membrane GP	Kim et al. (2019)
Resveratrol	Polyphenol	*In silico*	↓replication of influenza A virus	Fioravanti et al. (2012)
			↓JNK and p38MAPK, modulating TLR-9-induced IFN-β production	Li et al. (2015)
			↓inhibit H1N1 NA	Lin et al. (2015)
			↓H5N1 viral replication	Chen et al. (2012a)
			↓proinflammatory cytokines	Friel and Lederman, (2006)
			↓cytokine-induced oxidative stress	

## DENV

DENV is accompanied by encephalopathy, encephalitis, meningitis, myositis, myelitis, acute disseminated encephalomyelitis, neuromyelitis optica, optic neuritis (Prabhat et al., 2020), GBS, neuroophthalmic complications, intracranial hemorrhage, cerebral edema, hyponatremia, hypokalemia, and cerebral anoxia (Schlindwein et al., 2020). Several reports indicated various phytochemicals with antiviral activity against DENV such as quercetin, daidzein, naringin, hesperetin, glabranine, and 7-*O*-methyle glabranin (Zandi et al., 2011). In this regard, quercetin has been shown to inhibit dengue polymerase enzyme with an IC_50_ value of 3.6 μM and lead to the inhibition of DENV replication (Coulerie et al., 2014). Anusuya et al. showed that quercetin and similar structural phytochemicals have antiviral effects against DENV RdRp, based on an *in silico* study (Anusuya and Gromiha, 2017). In this line, Manjula and coworkers investigated that quercetagetin, quercetin, myricetin, and kaempferol have an anti-DENV effect against DENV NS5 methyltransferase RNA capping site by using an RNA intervention mechanism (Manjula and Kumaradhas, 2020). It has been shown that quercetin and fisetin have antiviral activities, confirmed by *in silico* evidence. As reported by Jasso-Miranda et al*.*, quercetin and fisetin interact with E, NS1, NS3, and NS5 proteins, thereby inhibiting a different process in the viral replicative cycle. Consequently, quercetin inhibited IL-6 and TNF-α and changed the level of IL-10 and IFN-γ in DENV-2 (Jasso-Miranda et al., 2019).

Luteolin as another natural compound inhibited DENV replication by blocking the later phase of DENV viral lifecycle in infected cells (Peng et al., 2017) and inhibiting the cellular proprotein convertase furin viral NS3 protease (Peng et al., 2018). Frabasile et al*.* have shown that naringenin can affect replication and/or maturation of the DENV life cycle, which leads to inhibition of viruses (Frabasile et al., 2017). As developed by Calvo et al., EGCG has antiviral effects against DENV, evaluated by docking studies in interacting with the function of proteins at multiple binding sites (Vázquez-Calvo et al., 2017). *In silico* evidence has shown that hirsutine (an alkaloid) has anti-DENV activity, as it interferes with the late phase of the DENV lifecycle (Hishiki et al., 2017).

Based on molecular docking evidence quercetagetin, kaempferol 3-*O*-rutinoside, rutin, hyperoside, and epicatechin can inhibit DENV RNA and lead to the reduction of replication and neurological sign (Dwivedi et al., 2020). It has been shown that DENV-2 induced IL-6, IFN-γ, and IL-10, and quercetin, naringin, catechin, and fisetin can change signaling pathways in the innate response to reduce neurological disorders being hopeful candidates for therapy of neurological disorders (Igbe et al., 2017).

It has been shown that ubiquitin-proteasome system reduced the structural E-protein that could affect DENV infection. In this line, curcumin can cause the accumulation of viral proteins and promote the accumulation of ubiquitin conjugated proteins, which reduces DENV infection (Padilla-S et al., 2014). Salidroside increases the level of double-stranded RNA-dependent protein kinase and phosphorylated eukaryotic initiation factor 2 (p-eIF2*a*), which leads to the suppressed synthesis of viral proteins and declined level of NF-κB, towards suppressing viral replication during the early phase of DENV infection (Loaiza-Cano et al., 2021).

Nordihydroguaiaretic acid has shown inhibitory effects on the DENV. Besides, this phenolic lignan and its methylated derivative inhibited flaviviruses by impairing viral replication regarding suppressing encephalopathy. Since the multiplication of flavivirus is highly dependent on the metabolism of host cell lipid, the antiviral effect of nordihydroguaiaretic acid is associated with its ability to disturb the lipid metabolism through interfering with the sterol regulatory element-binding proteins pathway (Merino-Ramos et al., 2017).

In general, various phytochemicals have the potential of being used against neuronal signs of DENV by suppressing associated dysregulated pathways (Table 2).

**TABLE 2:** Preclinical evidence on the use of candidate phytochemicals against DENV and related neuropharmacological responses.

Compounds	Classification	Type of study	Mechanisms	References
Betulinic acid	Triterpenoid	*In vivo*	↓entry phase of the viral replication cycle, modulating viral RNA replication and viral protein synthesis	Choy Loe et al. (2020)
EGCG	Catechin	*In silico*	Interact with the function of proteins at multiple binding sites	Vázquez-Calvo et al. (2017)
Fisetin	Flavonol	*In vivo*	Interact with E, NS1, NS3, and NS5 proteins	Jasso-Miranda et al. (2019)
			↓different process in the viral replicative cycle	
			↓IL-6 and TNF-α and changes the level of IL-10 and IFN-γ	
Hirsutine	Alkaloid	*In silico*	Interfere with late phase in DENV lifecycle	Hishiki et al. (2017)
Kaempferol	Flavonol	*In vivo*	Anti-DENV effect against DENV NS5 MTase RNA capping site	Manjula and Kumaradhas, (2020)
Luteolin	Flavone	*In vivo*	↓replication	Peng et al. (2018)
			↓later phase of DENV viral lifecycle in infected cells	
			↓cellular proprotein convertase furin viral NS3 protease	
Myricetin	Flavonoid	*In silico*	Double-stranded RNA interaction inhibition	Daino et al. (2018)
Naringenin	Flavonoid	*In vivo*	↓replication and/or maturation of the DENV life cycle which lead to inhibition of viruses	Frabasile et al. (2017)
Quercetin	Flavonol	*In vivo*	↓replication virus and antiviral effect against DENV RdRp	Coulerie et al. (2014)
			Interact with E, NS1, NS3, and NS5 proteins	
			↓different process in the viral replicative cycle	
			↓IL-6 and TNF-α and changes the level of IL-10 and IFN-γ in DENV-2	
Resveratrol	Polyphenol	*In vivo*	↓viral protein synthesis or transcription, modulating viral-related gene expressions or signaling	Han et al. (2017)
Salidroside	Phenylpropanoid glycoside	*In vivo*	↑PKR and P-eIF2*a*	Loaiza-Cano et al. (2021)
			↓synthesis of viral proteins	
			↓NF-κB	
			↓viral replication during the early phase	

## ZIKV

ZIKV is related to fetal malformations such as craniosynostosis, intrauterine growth restriction, craniofacial malformations, pulmonary hypoplasia, arthrogryposis, and severe ventriculomegaly secondary to midbrain damage with aqueduct atresia or stenosis; the occipital lobe sometimes acquires a cystic appearance and moderate ventriculomegaly with the presence of shallow grooves or agyria (De Melo Marques et al., 2019). Similar to other emerging infectious disease, ZIKV is affected by phytochemicals through different mechanisms to prevent associated neuronal signs. Loe et al*.* indicated that betulinic acid (a triterpenoid) suppressed the entry phase of the viral replication cycle and also can affect viral RNA replication and viral protein synthesis, as it has antiviral effects against other RNA viruses. One of them is ZIKV, a flavivirus related to DENV (Choy Loe et al., 2020). Mohd and coworkers observed that resveratrol inhibited the ZIKV and suppressed the early stage of virus entry into the host cells *via* inactivating the phosphorylation of the epidermal growth factor receptor (EGFR) (Mohd et al., 2019). Similarly, Suroengrit and colleges observed that chrysin has potent inhibitory effects on ZIKV (Suroengrit et al., 2017).

Recently, it has been suggested that quercetin and other derivatives can affect viral entry process viruses (Qiu et al., 2016) and suppress DENV-2 replication by inhibiting viral RNA polymerase (Fanunza et al., 2020). Consequently, another flavonoid EGCG has an antiviral effect against ZIKV as it inhibited the entry into the host cell and also can inhibit essential phases in the replication cycle viruses to bind the nucleoside-triphosphatase (NTPase) site in ZIKV (Kumar et al., 2020). Studies have also shown that EGCG and baicalin bind to the virus E-protein and can inhibit the entry of ZIKV into host cells (Wang et al., 2019). Also, baicalein and baicalin have antiviral activity against ZIKV infections (Oo et al., 2019). Another *in silico* evidence has shown curcumin, rutin, sanggenon, delphinidin, isoquercetin, naringenin, and EGCG as antiviral activity against the ZIKV NS2B-NS3 protease activity and can inhibit replication of the virus to reduce neurological sign (Mounce et al., 2017; Wong et al., 2017; Ahmed et al., 2020; Albuquerque de Oliveira Mendes et al., 2020; Yadav et al., 2021).

Evidence indicates that delphinidin and curcumin inhibited ZIKV infection *via* blocking the virus entry phase (Clain et al., 2019). As evaluated by a docking study, naringenin also inhibited ZIKV infection in human cells. It prevented NS2B-NS3 protease activity of ZIKV *via* the formation of hydrogen bonds between the phenol hydrogens of naringenin and amino acid of the virus protease (De Oliveira Mendes et al., 2020). Lim et al. investigated several other polyphenol compounds which belong to flavonols, flavanols, flavones, and flavanones such as luteolin, chrysin, myricetin, ampelopsin, astragalin, rutin, icaritin, hesperidin, pyrogallol, pyrocatechol, caffeine, gallic acid against ZIKV NS2B-NS3 proteases, and observed the potential of these compounds in the inhibition of ZIKV replication (Lim et al., 2017). In a molecular docking study, some flavonoids amentoflavone, fisetin, isorhamnetin, and theaflavin 3-gallate have shown potential inhibitory activity against ZIKV NS3-NS2B protease (Bhargava et al., 2019; Zou et al., 2020; Eberle et al., 2021; Lima et al., 2021; Yadav et al., 2021). Roy et al. based on *in silico* evidence indicated that several natural products such as myricetin, gossypol, naringenin, apigenin, luteolin, isorhamnetin, daidzein, resveratrol, and catechin inhibited Zika NS2B-NS3 *via* binding to a pocket on the active site and suppressed replication of ZIKV to be useful for the treatment of neurological diseases (Roy et al., 2017; Cataneo et al., 2019; Gao et al., 2019).

Silymarin and pinocembrin, as other natural compounds, also inhibit the production of ZIKV in the early phase (Le Lee et al., 2019; Da Silva et al., 2020). Evidence indicated that cephalotaxine (an alkaloid) has anti-ZIKV activity and disrupts the life cycle of ZIKV, and cephalotaxine has the potential to be developed as a therapeutic agent against ZIKV (Lai et al., 2020). Recently, it has been shown a polyphenol-rich extract from *Aphloia theiformis* (Vahl) Benn. can suppress ZIKV and DENV infection *via* inhibiting the virus entry phase (Clain et al., 2019).

Overall, by targeting ZIKV proteases, phytochemicals seem to be promising candidates in combating emerging infectious diseases. Additionally, modulation of TAM may be a critical way for phytochemicals to combat ZIKV.

## EBOV

Potential plant-derived secondary metabolites are shown to combat EVD. In this regard, curcumin blocks cytokine storm by suppressing IL-1, IL-6, and TNF-α, which correlates with clinical manifestations of Ebola (Sordillo and Helson, 2015). Additionally, bisdemethoxycurcumin, demethoxycurcumin, and tetrahydrocurcumin are other major metabolites of curcumin with proven antiviral activity. In this line, bisdemethoxycurcumin indicated maximum inhibition of Ebola viral proteins among the curcuminoids (Baikerikar, 2017).

Recently, quercetin and other derivatives have been identified as affecting the entry process of EBOV (Qiu et al., 2016). Another flavonoid derivative called quercetin 3-β-*O*-D-glucoside protected against Ebola *in vivo*. Moreover, it was shown that this quercetin derivative inhibited the early steps of viral entry (Qiu et al., 2016). The mechanism of action of quercetin was to restore the IFN-I signaling cascade by a direct interfere with EBOV VP24 binding to karyopherin-α and thereby restoring IFN gene transcription and phosphorylated signal transducer and activator of transcription 1 (STAT1) nuclear transport (Fanunza et al., 2020).

Other studies indicated that genistein has antiviral effects and inhibited EBOV replication (Kolokoltsov et al., 2012). Ellagic acid, myricetin, and some other phytochemicals (Setlur et al., 2017) as anti-Ebola compounds act through inhibiting virus entry (Cui et al., 2018) and RNA interaction, respectively (Daino et al., 2018). An *in vitro* antiviral effect of oleandrin acts as a novel cardiac glycoside against the EBOV (Newman et al., 2020). Some preclinical evidence suggests the effectiveness of cannabinoids in viral diseases (Mabou Tagne et al., 2020). Altogether, phytochemicals have shown a promising future against EVD.

**FIGURE 2:**
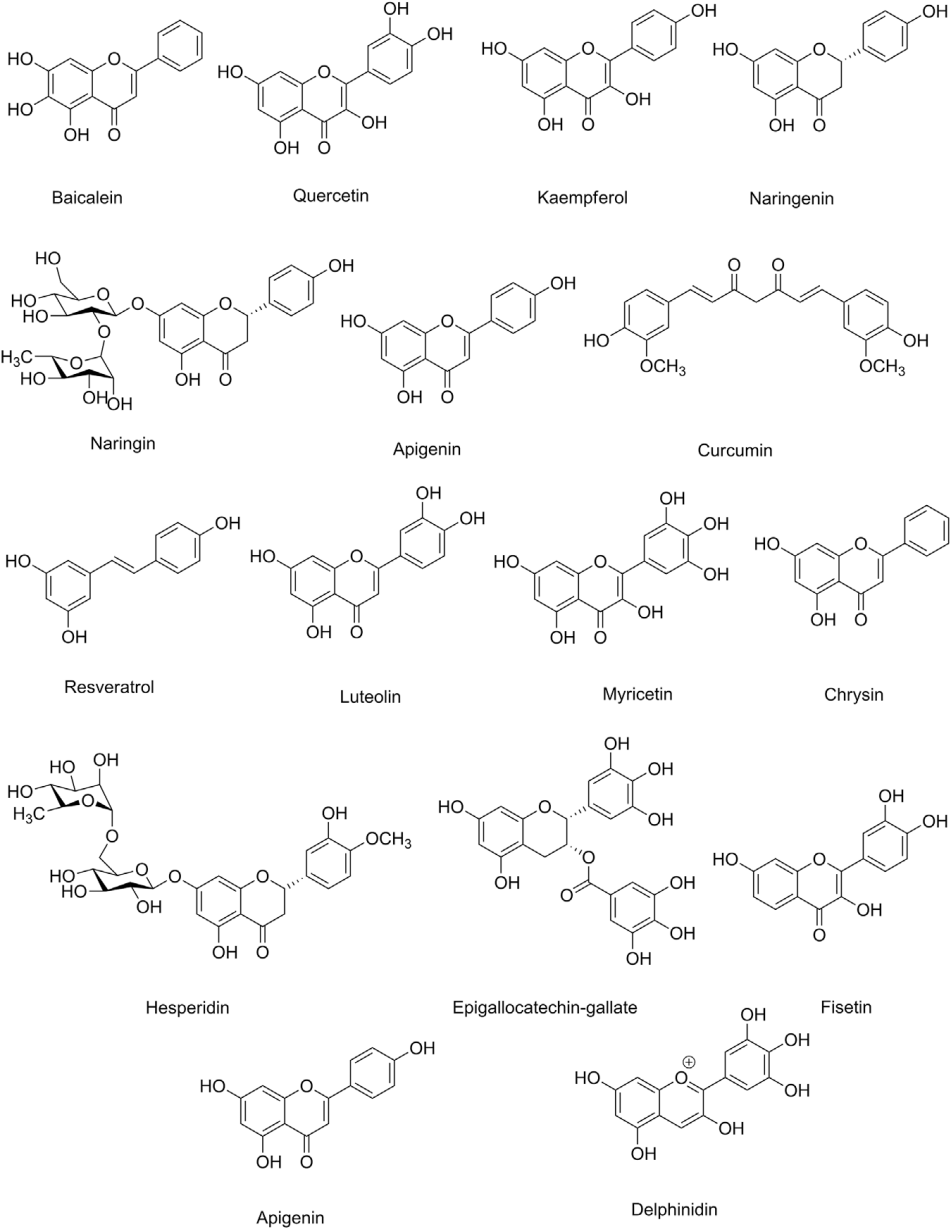
Selected chemical structures of phenolic compounds in combating neuronal signs of emerging infectious diseases.

**FIGURE 3:**
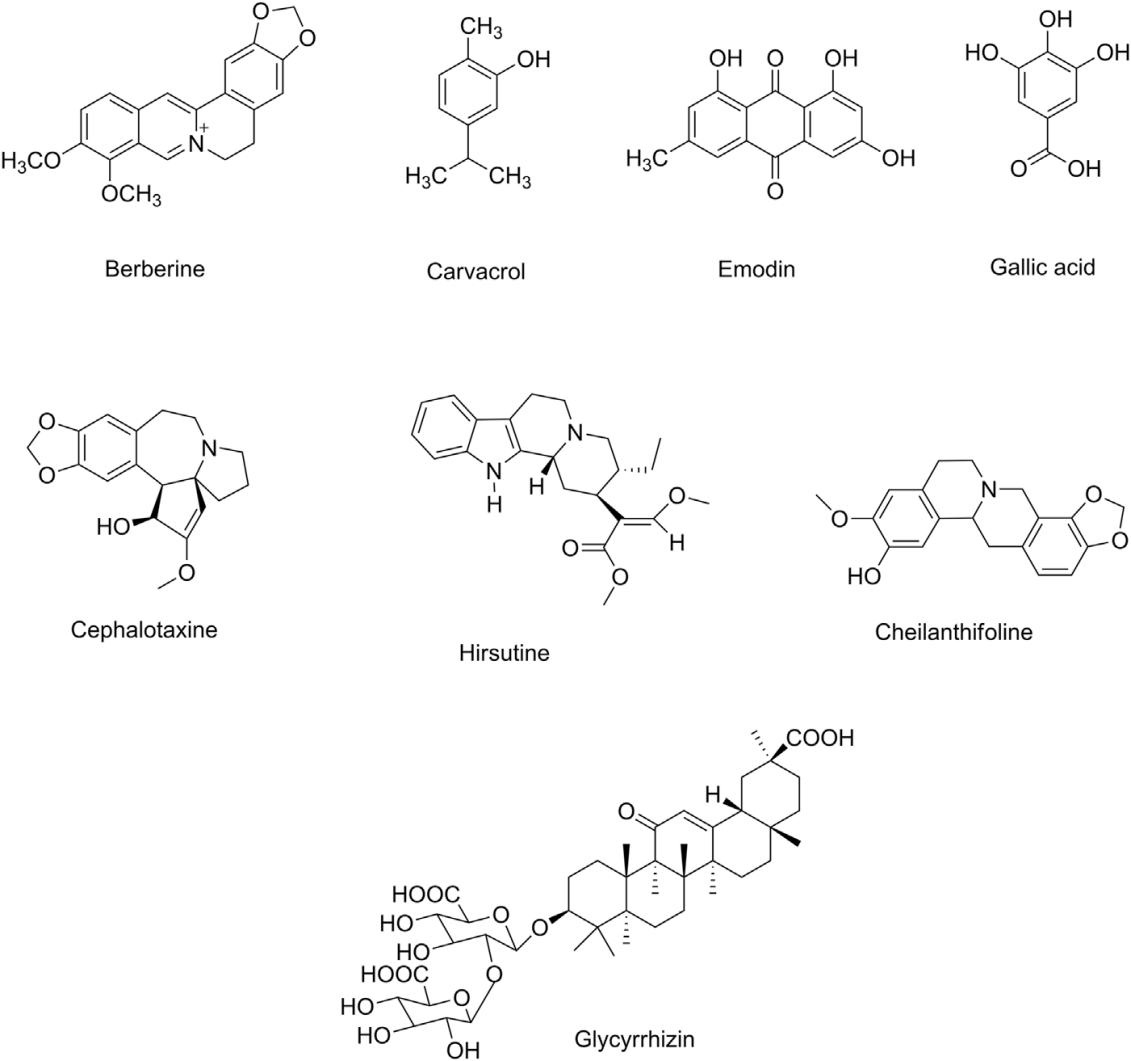
Selected chemical structures of alkaloids and miscellaneous compounds in combating neuronal signs of emerging infectious diseases.

Table 3 indicates the preclinical evidence on the use of candidate phytochemicals against ZIKV and EBOV and related neuronal manifestations. Figure 2 and Figure 3 indicate selected chemical structures of phenolic compounds, alkaloids, and miscellaneous compounds in combating neuronal signs of emerging infectious diseases.

**TABLE 3:** Some preclinical evidence on the use of candidate phytochemicals against ZIKV and EBOV and neuropharmacological responses.

Disease	Compounds	Classification	Type of study	Mechanisms	References
Zika virus	Curcumin	Polyphenol	*In vivo*	↓virus entry phase	Ahmed et al. (2020)
					Yadav et al. (2021)
	EGCG	Catechin	*In vivo*	↓entry into the host cell	Kumar et al. (2020)
				↓essential phase in the replication cycle of viruses; bind to NTPase site	
	Naringenin	Flavonoid	*In vivo*	↓NS2B-NS3 protease activity *via* the formation of hydrogen bonds between the phenol hydrogens of naringenin and amino acid of the virus protease	Roy et al. (2017)
					Cataneo et al. (2019)
					Gao et al. (2019)
Ebola virus	Curcumin	Polyphenol	*In vivo*	↓cytokine release, such as IL-1, IL-6, and TNF-α	Sordillo and Helson, (2015)
	Ellagic acid	Polyphenol	*In vivo*	Anti-Ebola virus, entry inhibition	Cui et al. (2018)
	Genistein	Isoflavone	*In vivo*	↓EBOV replication	Kolokoltsov et al. (2012)
	Quercetin	Flavonol	*In vivo*	↓entry process	Qiu et al. (2016)
	Quercetin 3-β-*O*-D-glucoside	Flavonoid	*In vivo*	↓early steps of viral entry	Qiu et al. (2016)

## Conclusion

**FIGURE 4:**
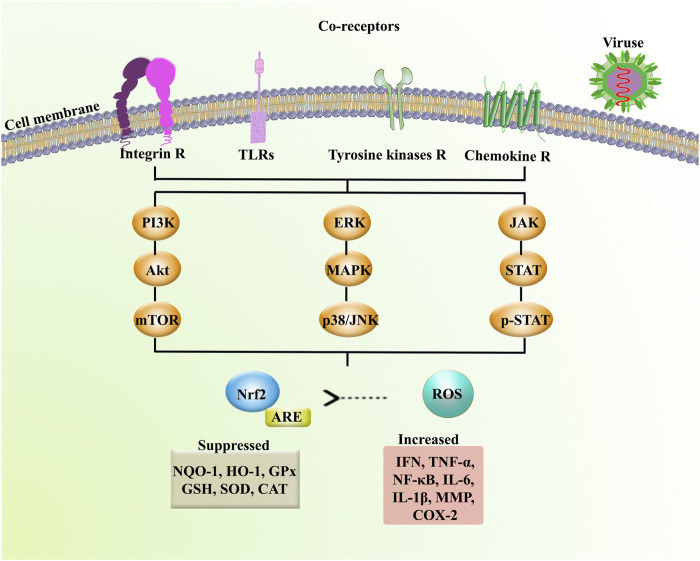
Receptors and signaling mediators involved in neuronal signs of emerging infectious diseases. Akt: protein kinase B, ARE: antioxidant response element, CAT: catalase, Chemokine R: chemokine receptor, COX-2: cyclooxygenase-2, ERK: extracellular signal-regulated kinase, GPx: glutathione peroxidase, GSH: glutathione, HO-1: heme oxygenase-1, IFN: interferon, IL: interleukin, Integrin R: integrin receptor, JAK: Janus kinase, mTOR: mammalian target of rapamycin, MMP: matrix metalloproteinase, NF-κB: nuclear factor-kappa B, NQO-1: NAD(P)H Quinone Dehydrogenase 1, Nrf2: nuclear factor erythroid 2-related factor 2, p38: p38 mitogen-activated protein kinase, p-STAT: phospho-signal transducer and activator of transcription, SOD: superoxide dismutase, STAT: signal transducer and activator of transcription, TLRs: toll-like receptors, TNF-α: tumor necrosis factor-α, and Tyrosine kinase R: tyrosine kinase receptor.

Phytochemicals have been excellent sources of alternative therapeutic agents and lead compounds in combating viral diseases. As a top global priority, novel plant-derived antiviral agents are promising candidates in combating neuronal manifestations of emerging infectious diseases. Consequently, inflammatory/oxidative pathways are critical targets, including ILs, TLR, NF-κB, MAPK, iNOS, and AQP, as well as several enzymes involved in the virus life cycle (Figure 4). The potential of phytochemicals in demonstrating antiviral effects through inhibiting the viral life cycle indicates a promising future to find novel antiviral lead compounds against neuronal signs of emerging viral diseases. Previously, we have analyzed the capability of these phytochemicals in passing BBB towards neuroprotective responses (Fakhri et al., 2020b), which showed hopeful results for most plant-derived secondary metabolites in modulating the aforementioned dysregulated pathways in CNS. Additionally, to drawback, the pharmacokinetic limitation of phytochemicals, novel drug delivery systems, could pave the road towards neuroprotection against emerging viral diseases. In our previous study, we also provided that the potential of flavonoids, terpenes/terpenoids, chalcones, and alkaloids has been shown in targeting angiotensin-converting enzyme 2 (ACE2) and spike proteins against neurological signs of coronavirus disease 2019 (COVID-19) and found promising results in combating pathophysiological mechanisms of the virus.

**FIGURE 5:**
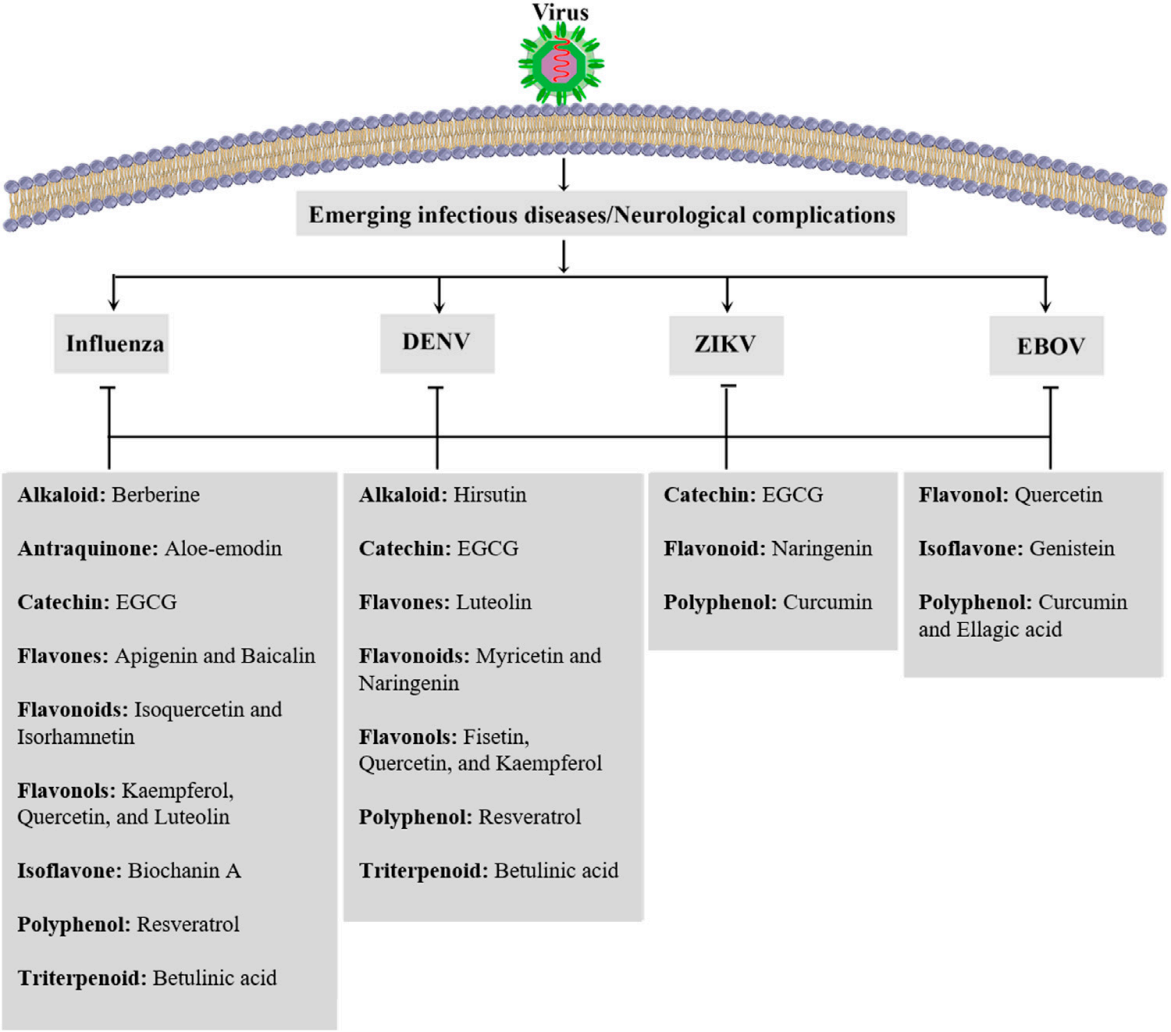
Candidate phytochemicals in combating neurological signs of emerging infectious diseases. DENV: dengue virus, EBOV: Ebola virus, EGCG: epigallocatechin gallate, and ZIKV: Zika virus.

In the present review, a mechanistic approach has been employed on plant-derived antiviral compounds with related pharmacological mechanisms, as alternative therapies against neuronal signs of emerging infections (Figure 5). Future research areas should include additional *in vitro* and *in vivo* experimentation to highlight the significant pathophysiological mechanisms of viral diseases. Introducing other effective and novel plant-derived antiviral lead compounds through modulating such dysregulated pathways could pave the road in combating viral infections. It should be followed by well-controlled clinical trials to assess phytochemicals as multi-target alternative agents. Such reports will help reveal more applications of phytochemicals in the prevention, management, and treatment of emerging viral diseases.
